# Comparison of utility measures and auditory performance in children with cochlear implants and hearing aids in India: a cross-sectional study

**DOI:** 10.1016/j.bjorl.2026.101776

**Published:** 2026-03-19

**Authors:** Anuradha Sharma, Shankar Prinja, Ravinder Thakur, Rajwinder Kaur, Sanjay Munjal, Jaimanti Bakshi

**Affiliations:** a Post Graduate Institute of Medical Education and Research, Department of Otolaryngology, Speech and Hearing Unit, Chandigarh, India; b Post Graduate Institute of Medical Education and Research, Department of Community Medicine, Chandigarh, India

**Keywords:** Cochlear implant, Health utilities, Quality of life, PEACH, TEACH

## Abstract

**Objectives:**

Hearing loss significantly impacts auditory function and quality of life in children. Assessing Health-Related Quality of Life (HRQoL) using utility measures provides valuable insight into the effectiveness of interventions such as Cochlear Implants (CI) and Hearing Aids (HA). To compare HRQoL using multiple utility measures (HUI-3, EQ-5D-5 L, VAS) and functional auditory performance (PEACH, TEACH) in children with cochlear implants, hearing aids, and unaided severe to profound hearing loss.

**Methods:**

Ninety children aged 7–10 years were divided into three groups (n = 30 each): CI users, HA users, and children with unaided severe to profound hearing loss. Utility measures and auditory performance scales were administered. Statistical analysis involved ANOVA and Pearson’s correlation.

**Results:**

CI users exhibited the highest utility scores (HUI-3 = 0.68964; EQ-5D = 0.88446) and auditory performance scores (PEACH = 37; TEACH = 25.83). Strong correlations were found between utility scores and auditory performance. HUI-3 showed greater sensitivity in detecting hearing-specific QOL improvements.

**Conclusion:**

Cochlear implantation results in superior HRQoL and auditory performance compared to hearing aids. Use of sensitive, hearing-specific tools like HUI-3 is recommended.

**Level 3:**

Non-randomized controlled cohort/follow-up study.

## Highlights

• First Indian study to evaluate long-term QOL outcomes in CI and HA children.
• Multi-tool analysis reveals cochlear implants greatly outperform hearing aids.
• HUI-3 effectively captures hearing-related QOL gains missed by EQ-5D.
• Functional auditory outcomes strongly linked to improved health utility scores.
• Robust evidence supporting CI as the superior intervention for paediatric deafness.

## Introduction

The multifaceted notion of Health-Related Quality of Life (HRQoL), which includes social, emotional, and physical well-being, offers important insights into the wider effects of disorders like hearing loss on individuals' day-to-day lives.^1^^,^^2^ HRQoL is evaluated using utility evaluations which facilitates comparisons between treatments and helps with healthcare budget allocation alternatives. Cost-utility studies, which combine quality and quantity of life to generate Quality-Adjusted Life Years (QALYs), rely heavily on utility evaluations, which range from 0 (death) to 1 (perfect health).^3^^,^^4^ The analysis of cost effectiveness may be done using this utility score. Quality-Adjusted Life Years (QALYs) are used in Cost Utility Analyses (CUAs) to quantify the effectiveness of therapy.^5^ When Quality of Life (QoL) and quantity of life are combined, a QALY is generated.^6^ Patients with hearing loss have had their utility and quality of life assessed using a variety of instruments, including the Health Utilities Index Mark 3 (HUI3), the EuroQol Five-Dimensional Questionnaire (EQ-5D), the Short Form 38 health survey (SF-36), the Visual Analogue Scale (VAS), the Time Trade-Off (TTO), and disease-specific evaluations like the PEACH and TEACH scales. Inconsistencies across these approaches, however, may result in different cost-effectiveness estimates.^7^, ^8^, ^9^

Very few studies have used different utility methods to compare the outcomes of cochlear implantation. The results of this study showed that there were significant differences in the utility gains and health utility scores ascertained by each of the instruments.^10^, ^11^, ^12^, ^13^, ^14^ According to the results, HUI3 should be preferred for QOL evaluation after the intervention because patients report higher utility scores when using the HUI3 questionnaire than when using other instruments^12^^,^^13^ and it is less likely to overestimate the cost-utility of a second Cochlear implant.^10^

This study aimed to fill this gap by comparing utility measures and auditory performance in unilateral cochlear implant users and bilateral hearing aid users after four years of intervention. Few studies have compared the utility outcomes of Cochlear Implantation (CI) versus Hearing Aid (HA) usage, particularly in the Indian context where cultural, linguistic, and socioeconomic diversity can influence outcomes. Moreover, the lack of a gold standard utility instrument for economic evaluation at this time necessitates a deeper comprehension of each instrument in light of the circumstances of hearing disability. Given the ongoing disagreements around the surgery, as well as the fact that it is an expensive intervention, this review is essential.^14^ This will also shed light on how the instruments used to gauge QOL were designed. This is necessary to show that HRQL instruments are measuring the things they are supposed to measure, which necessitates empirical data.^15^ The current study aimed to evaluate PEACH and TEACH scores in the same group, compare utility scores obtained from HUI3, EQ-5D, and VAS in CI and HA users following four years of intervention, and investigate relationships between utility measures and auditory performance.

## Methods

### Study design and settings

In order to evaluate and compare health-related quality of life and auditory performance across three groups of children with severe to profound hearing loss, a cross-sectional comparative research was carried out in the speech and hearing divisions of a tertiary care hospital in North India. Participants in the study were patients who came to the speech and hearing unit for auditory or listening training or because they complain of hearing loss.

### Participants and inclusion criteria

Out of the 132 people that underwent screening, 90 met the criteria established for the study. Ninety children between the ages of 7 and 10 were enlisted and sorted into three groups (n = 30 each): children with unaided Severe-to-Profound Hearing Loss (SPHL), unilateral CI users, and bilateral HA users. >4-years of device use and free-field thresholds <40 dB HL from 250 Hz to 4000 Hz were essential for enrolment. Auditory neuropathy, retrocochlear disease, additional impairments, and inner ear anomalies were among the exclusion criteria. Only unilateral implantees were included in the Cochlear implant group. The hearing aid group did, however, contain individuals who had bilateral aids.

### Instruments

The quality of life was evaluated using the Visual Analogue Scale (EQ-VAS), the European Quality of Life Questionnaire (EQ-5D-Y), Euro-QOL, and the Health Utilities Index Mark 3 (HUI3).

#### HUI3

Eight facets of health are evaluated by the HUI3, a thorough verified self-report questionnaire that includes vision, hearing, speech, ambulation, dexterity, emotion, cognition, and pain. There were up to six layers in each dimension. Utility scores are assigned using the HUI3 rating approach on a scale of 1.00 (perfect health) to 0.00 (death).^16^

#### EQ-5D

A self-administered questionnaire, the EQ-5D evaluates five dimensions: mobility, self-care, normal activities, pain/discomfort, and anxiety/depression. Each dimension or domain has three possible scores: none, substantial, and intense.

A five-digit number (11 111, 11 112, etc.) that corresponds to a series of answers to the five dimensions is the EQ-5D-5L health status. Each digit indicates the number of issues in the corresponding dimension. The EQ-5D-5L was utilised to create a single utility score between 0 and 1 based on respondents' answers to questions on how audiological rehabilitation affected their life. Excellent health was denoted by a utility value of 1, while death was signified by a score of 0. One value for the health-status index is produced by this instrument.^17^^,^^18^

Additionally, the EuroQoL VAS was used to ask each patient to rate their present state of health on a scale of 0 to 100. It is composed of a vertical line with clear ends that is 20 centimetres long. The ratings, as seen by the patients, are ordinal rankings of health outcomes, where '0' indicates the poorest health status and '100' the greatest.

#### Auditory performance tools

Parents’ Evaluation of Aural/Oral Performance of Children (PEACH) and Teachers’ Evaluation of Aural/Oral Performance of Children (TEACH).

The assessment of children's oral and auditory performance is done by both parents (PEACH) and teachers (TEACH). The PEACH rating scale is based on parental observation and consists of 13 questions. Responses are scored on a five-point scale. The first two questions asked about your feelings when wearing hearing aids and in noisy environments. The effectiveness of amplification in neonates and children with hearing impairments was evaluated using this scale. Similar to this, TEACH is a rating system based on observations of teachers that consists of eleven questions. Responses are scored on a five-point scale.

### Procedure

Parents completed the validated Hindi versions of the questionnaires with clinician assistance when needed. The clinician rephrased the question as to whether parents could understand or infer the meaning of any question. All gathered data were coded and entered into an Excel spreadsheet. Finally, the Excel spreadsheet was exported into SPSS software for data analysis.

### Statistical analysis

To compile demographic information, descriptive statistics were employed. Mean scores were compared using Tukey's post-hoc tests and one-way ANOVA. The associations between auditory performance and quality-of-life metrics were investigated using Pearson's correlation. ANOVA's assumptions were studied.

## Results

### Participant demographics

Ninety patients were included in the study, of which 46 (51.11%) were male and 44 (48.88%) were female. The mean chronological ages were 8.6 (CI), 8.2 (HA), and 9.4 years (SPHL). Hearing ages for CI and HA groups were 4.6 and 4.2 years respectively. The gender distribution was balanced across groups (Table 1).

**Table 1:** Demographic characteristics of the patients included in the study.

	Cochlear implant	Hearing aid group	Severe-Profound hearing loss group
Total n = 30	Male ‒ 12	Male ‒ 14	Male ‒ 20
	Female ‒ 18	Female ‒ 16	Female ‒ 10
Chronological age	Mean age ‒ 8.6 years	Mean age ‒ 8.2 years	Mean age ‒ 9.4 years
Hearing age	Mean age ‒ 4.6 years	Mean age ‒ 4.2 years	Mean age ‒ 0

Health utility scores

**Fig. 1:**
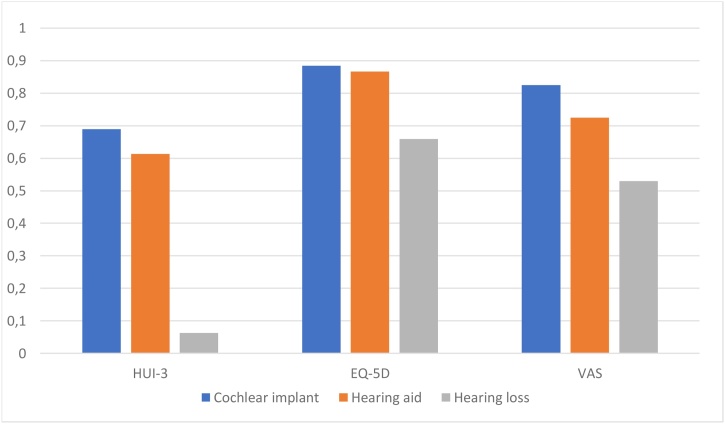
Mean utility score in the CI, HA, and hearing loss groups using HUI3, EQ-5D, and EQ-VAS tools.

The mean HUI3 scores for the cochlear implant group, hearing aid users, and severe-profound hearing loss group were 0.68964, 0.6138, and 0.06339, respectively. The mean EQ-5D scores for cochlear implanted group, hearing aid users, and severe-profound hearing loss groups were 0.8844, 0.8676, and 0.6595, respectively (Fig. 1, Table 2).

**Table 2:** Mean scores and F-values of generic QOL and auditory performance evaluation tools.

Parameter	Group	n	Mean	Std. Deviation	F-statistic
HUI Final Score	CI	30	0.68964	0.10596	365.214*
	Hearing aid	30	0.61385	0.13208	
	Profound	30	0.06339	0.01069	
	Total	90	0.45563	0.29688	
EQ-5D	CI	30	0.88446	0.01836	259.095*
	Hearing aid	30	0.86767	0.02421	
	Profound	30	0.65958	0.06729	
	Total	90	0.80390	0.11115	
VAS Scores	CI	30	820.50000	30.55983	251.592*
	Hearing aid	30	720.50000	60.53241	
	Profound	30	530.00000	50.01721	
	Total	90	690.33333	130.34082	
Peach-Total	CI	30	370.00000	10.78113	4368.894*
	Hearing aid	30	260.16667	20.06920	
	Profound	30	0.00000	0.00000	
	Total	90	210.05556	150.69610	
Teach Scores	CI	30	250.83333	10.05318	3333.075*
	Hearing aid	30	200.36667	10.97368	
	Profound	30	0.00000	0.00000	
	Total	90	150.40000	110.25077	

* denotes values that are statistically significant at p < 0.05.

### Auditory performance scores

**Fig. 2:**
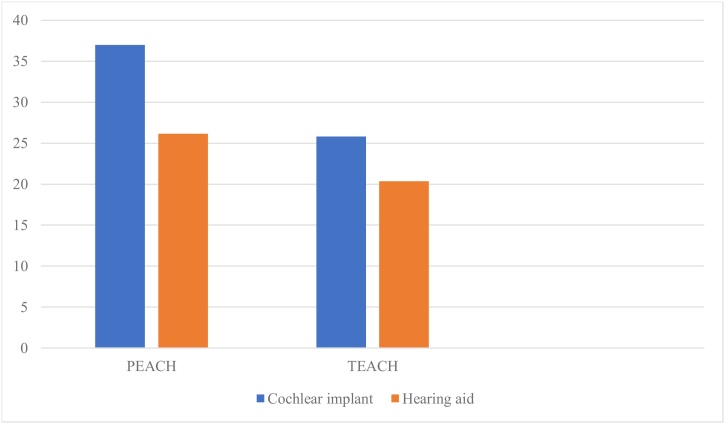
Mean PEACH and TEACH scores for cochlear implant and hearing aid users.

The mean PEACH scores for the cochlear implant group and hearing aid user group were 37 and 26.1, respectively. The mean TEACH scores for cochlear implanted group and users’ group were 25.83 and 20.36, respectively. SPHL group scored zero on both scales (Fig. 2).

Comparison of quality-of-life measures and auditory performance evaluation scores among the three groups

One-way ANOVA results showed a significant difference across all groups for all measures (p < 0.01). The highest significant difference was seen in P.E.A.C.H scores, while the least significant difference was shown in VAS scores. The results of the one-way ANOVA are shown in Table 2.

### Multiple comparison test

Post-hoc Tukey's tests confirmed significant differences in the mean scores of HUI3, VAS, P.E.A.C.H., and T.E.A.C.H. measures between SPHL and both intervention groups for all measures. However, no significant difference was observed between CI and HA users in the EQ-5D (Table 3, Table 4).

**Table 3:** Post-hoc analysis results of HUI3, EQ-5D, and VAS scores across the three groups.

Tukey-B									
		HUI3			EQ-5D		VAS		
		Subset for α = 0.05			Subset for α = 0.05		Subset for α = 0.05		
Group	n	1	2	3	1	2	1	2	3
Profound	30	0.06339			0.65985		53.000		
Hearing aid	30		0.61385			0.86767		72.500	
CI	30			0.68964		0.88446			82.5000

**Table 4:** Post-hoc analysis results of auditory performance scales across the three groups.

Tukey-B							
		P.E.A.C.H Scores			T.E.A.C.H Scores		
		Subset for α = 0.05			Subset for α = 0.05		
Group	n	1	2	3	1	2	3
Profound	30	0.00			0.000		
Hearing aid	30		26.16667			20.36667	
CI	30			37.000			25.8333

### Correlations

Correlation between the QOL measures and auditory performance evaluation measures: Pearson’s correlation test showed strong positive correlations between utility measures and auditory performance (r > 0.7) in both the CI and HA groups, with the strongest correlations in the CI group; the results of the Person’s correlation test are shown in Table 5, Table 6, Table 7.

**Table 5:** Pearson’s correlation coefficient values for various tools in cochlear implanted group.

	Correlations				
	HUI Final Score	EQ-5D	VAS Scores	Peach-Total	Teach Scores
HUI Final Score	1.0000	0.8842*	0.9297*	0.9446*	0.9061*
EQ-5D		1.0000	0.7910*	0.8393*	0.8094*
VAS Scores			1.0000	0.9248*	0.9294*
Peach-Total				1.0000	0.9724*
Teach Scores					1.0000

* denotes values that are statistically significant at p < 0.05.

**Table 6:** Pearson’s correlation coefficient values for various tools in hearing aid group.

	Correlations				
	HUI Final Score	EQ-5D	VAS Scores	Peach-Total	Teach Scores
HUI Final Score	1.0000	0.8302*	0.7671*	0.9444*	0.8841*
EQ-5D		1.0000	0.7138*	0.6981*	0.5761*
VAS Scores			1.0000	0.7831*	0.7036*
Peach-Total				1.0000*	0.9559*
Teach Scores					1.0000

* denotes values that are statistically significant at p < 0.05.

**Table 7:** Pearson’s correlation coefficient values for various tools in severe-profound hearing loss group.

	Correlations		
	HUI Final Score	EQ-5D	VAS Scores
HUI Final Score	1.0000	0.5836*	0.6532*
EQ-5D		1.0000	0.8022*
VAS Scores			1.0000

* denotes values that are statistically significant at p < 0.05.

The correlation between any two parameters was maximum in the cochlear-implanted group. This shows that all pairs of parameters are significantly positively correlated in the cochlear-implanted group.

## Discussion

To the best of our knowledge, this is the first study from India to measure Health-Related Quality of Life (HRQoL) using multiple utility-based instruments (HUI3, EQ-5D-5 L, VAS) alongside functional auditory performance tools (PEACH and TEACH) in children with unilateral Cochlear Implants (CI) and bilateral Hearing Aids (HA) after four years of intervention. Our findings provide robust evidence that both cochlear implantation and hearing aid use significantly enhance the quality of life and auditory performance in children with severe to profound hearing loss, as compared to those without any amplification.

The mean utility scores observed in our study (EQ-5D-5L: 0.88446 for CI users, 0.86767 for HA users; HUI3: 0.68964 for CI users, 0.61385 for HA users) were comparable to those reported in previous international studies. Ramakers et al. (2016) reported that cochlear-implanted adults had higher utility scores using HUI3 than EQ-5D, reinforcing our finding that HUI3 is more sensitive to auditory-specific improvements.^12^ Similarly, Lee et al. (2006) demonstrated that cochlear implantation leads to higher cost-utility gains than hearing aids when measured using appropriate tools sensitive to auditory outcomes.^13^ Our results corroborate these observations by demonstrating that the inclusion of hearing- and speech-specific domains in HUI3 offers superior sensitivity over EQ-5D, which primarily focuses on general health dimensions without addressing communication-specific challenges.

Notably, our study uniquely highlights that in the Indian context, where linguistic diversity, socioeconomic disparities, and access to rehabilitation services vary significantly, both CI and HA users showed marked improvements in psychological well-being, social participation, and auditory performance. This multidimensional benefit aligns with the findings of Arnoldner et al. (2014), who showed that cochlear implants not only enhance auditory perception, but also improve overall mental health and social integration.^11^ Our use of the PEACH and TEACH scales adds a valuable functional dimension to the assessment, reflecting real-world listening abilities both at home and in academic settings, an aspect that is often neglected in cost-utility studies.

The present study demonstrated statistically significantly higher scores in QOL and auditory performance for both CI and HA users compared to unaided children. The highest F-value observed in the PEACH scores underscores the efficacy of the tool in capturing the functional gains after auditory rehabilitation. These findings are supported by Sach (2002), who emphasized that beyond audiological thresholds, quality of life and social engagement should be key metrics for evaluating the success of cochlear implantation.^14^

The PEACH and TEACH scores in our study confirmed that cochlear implants offer superior functional outcomes compared to hearing aids. The mean PEACH score for CI users (37) was significantly higher than that for HA users (26.1), echoing the findings of Smulders et al. (2016), who reported enhanced auditory behaviors and environmental awareness in children with cochlear implants.^19^ Furthermore, the strong correlations observed between auditory performance measures and utility scores align with findings from studies by Barton et al. (2005) and Kuthubutheen et al. (2015), which indicate that better auditory performance translates directly into higher utility valuations and improved life satisfaction.^4^^,^^10^

An important insight from our research is the limited sensitivity of EQ-5D in differentiating between CI and HA users. This limitation mirrors the observations by Turner et al. (2013), who highlighted that EQ-5D often fails to detect subtle yet clinically significant differences in sensory domains.^9^ The lack of hearing- and speech-specific domains in EQ-5D reduces its utility in capturing the nuanced benefits of audiological rehabilitation, thereby reinforcing the argument for adopting multi-attribute instruments such as HUI3 in such contexts. Therefore, our study supports the growing body of evidence advocating tailored utility measurement tools in cost-effectiveness analyses of hearing interventions.

Another unique contribution of this study lies in its use of both parent (PEACH) and Teacher (TEACH) assessments, which reflect the child's auditory performance in diverse listening environments. This dual-perspective approach offers a holistic view of the child’s functional improvements, which is particularly valuable in resource-limited settings, where formal speech and language assessments may not always be feasible. This methodological addition distinguishes our study from prior work, which predominantly focused on either clinical measures or parental reports alone.

The observed positive correlations between utility scores and auditory performance highlight the intrinsic link between hearing-related functional gain and perceived quality of life. In our study, children with cochlear implants consistently demonstrated higher correlation coefficients across measures, indicating that the auditory advantages conferred by cochlear implants translate into broader psychosocial and quality-of-life benefits. This observation echoes the findings of Heintz et al. (2012), who noted that multidomain improvements following sensory interventions are essential drivers of increased utility scores.^7^

Our study also adds to the limited Indian literature on the cost-utility and quality-of-life outcomes of pediatric hearing loss rehabilitation. These findings are particularly relevant given the rising number of cochlear implantations in India under government-supported schemes, making it crucial to have locally generated evidence for policy and funding decisions.

## Limitations and future directions

The main limitation of the current study was the small sample size, and the impact of socioeconomic factors on QOL was not considered. It is recommended that future research should include a normal hearing group and disease specific QOL measure to evaluate the relationship between various tools to assess the outcomes of audiological rehabilitation.

## Conclusion

Cochlear implantation yields superior quality of life and auditory performance outcomes compared to hearing aids in children with severe to profound hearing loss. The study underscores the importance of using sensitive, hearing-specific utility instruments, such as HUI3, for a more accurate assessment of intervention benefits. The integration of functional auditory scales, such as the PEACH and TEACH, provides a comprehensive view of rehabilitation success. Our findings advocate for the inclusion of such measures in both clinical and economic evaluations of auditory rehabilitation programs in India.

## ORCID ID

Shankar Prinja: 0000-0001-7719-6986

Ravinder Thakur: 0009-0006-3844-1241

Rajwinder Kaur: 0009-0007-7575-7832

Jaimanti Bakshi: 0000-0003-4412-7620

## CRediT authorship contribution statement

Conceptualization: Dr Anuradha, Dr Shankar Prinja. Data curation: Dr Anuradha. Formal analysis: Dr Anuradha, Dr jaimanti Bakshi. Funding Acquisition: Dr Anuradha. Investigation: Mr Ravinder thakur, Ms Rajwinder kaur. Methodology: Dr Anuradha and Dr jaimanti bakshi. Project Administration: Dr Anuradha, Mr Ravinder thakur and Ms Rajwinder kaur. Resources: Dr Anuradha, Mr Ravinder thakur and Ms Rajwinder kaur. Software: None. Supervision: Dr Anuradha, Dr Shankar Prinja. Validation: None. Visualization: Dr Anuradha, Dr Shankar Prinja. Writing-original draft: Dr Anuradha, Mr Ravinder thakur, Ms Rajwinder kaur. Writing-review and editing: Dr Sanjay Munjal and Dr Jaimanti Bakshi.

## Disclosure

The Authors hereby certify that the work shown here is genuine, original and not submitted anywhere, either in part or full. All the necessary permissions from the patient, hospital and institution have been taken for submitting to your esteemed journal.

## Funding

This Project received specific grants from Department of Health Research (DHR), Indian Council of Medical Research (ICMR), New Delhi, India.

## Data availability statement

Data supporting the results will be available upon request from the corresponding author. The data is not accessible to the public because of confidentiality and ethical restrictions.

## Declaration of competing interest

There is no conflict of interest. I had full access to all of the data in this study and I take complete responsibility for the integrity of the data and the accuracy of the data analysis.
